# An Extremely Rare Case of Poorly Differentiated Thyroid Carcinoma Arising in Malignant Struma Ovarii and Concurrent Papillary Thyroid Carcinoma: A Case Study

**DOI:** 10.7759/cureus.44481

**Published:** 2023-08-31

**Authors:** Jessie C Harrison, Matthew Figh, Heather Shah

**Affiliations:** 1 Research, Alabama College of Osteopathic Medicine, Dothan, USA; 2 General Surgery, Decatur Morgan Hospital, Decatur, USA; 3 Hematology and Oncology, Clearview Cancer Institute in Alabama, Decatur, USA

**Keywords:** pax-8, thyroglobulin, mature cystic ovarian teratoma, monodermal teratoma, interprofessional education and collaboration, immunohistochemical markers, thyroid papillary carcinoma, poorly differentiated thyroid carcinoma, malignant struma ovarii

## Abstract

Ovarian tumors can be classified by their origin - epithelial tumors, germ cell tumors, and stromal tumors. Malignant struma ovarii (MSO) are 0.01% of all ovarian tumors. In order to be classified as a struma ovarii, more than 50% of the teratoma consists of thyroid tissue. The thyroid tissue in the struma ovarii exhibits the same histological and physiological properties as that of the cervical thyroid tissue. Poorly differentiated thyroid carcinoma (PDTC) is an extremely rare occurrence when arising from an MSO. Including this case report, there are only 10 reports of PDTC in the setting of MSO. Of these cases, this patient is the only one who presented with concurrent primary thyroid carcinoma (PTC). This case study examines how invaluable intra-professional collaboration is for appropriate diagnosis, along with attention to detail of identifying markers in pathology sections and use of the appropriate immunohistochemical analysis.

## Introduction

Ovarian tumors can be classified by their origin - epithelial tumors, germ cell tumors, and stromal tumors [1]. Germ cell tumors make up 15% of ovarian tumors, struma ovarii constitute 1% of ovarian tumors, and malignant struma ovarii are 0.01% of all ovarian tumors [2]. In order to be classified as a struma ovarii, more than 50% of the teratoma consists of thyroid tissue [3]. Though struma ovarii is rare, it is the most common type of monodermal teratoma - a germ cell tumor that is exclusively or predominantly derived from a single embryonic layer [4]. Struma ovarii are considered malignant if one of the following criteria is fulfilled: (1) there is extraovarian spread upon presentation, (2) ovarian serosa is infiltrated by the tumor on the surface, or (3) recurrence after initial surgery [1]. This report presents a case of an extremely rare case of poorly differentiated thyroid carcinoma (PDTC) in the setting of malignant struma ovarii (MSO) and a concurrent primary papillary thyroid carcinoma (PTC). The goal of this case study is to highlight the importance of intra-professional collaboration for appropriate diagnosis, along with attention to detail of identifying markers in pathology sections and use of the appropriate immunohistochemical analysis.

## Case presentation

**Figure 1:**
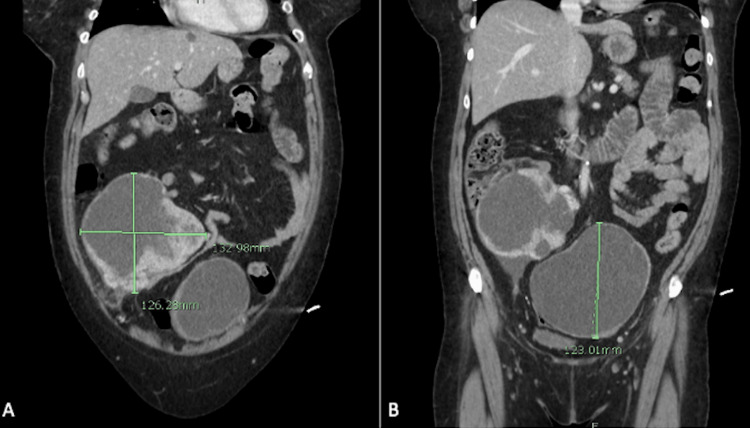
Coronal images of CT scan showing bilateral ovarian masses. A) Depicts the poorly differentiated thyroid carcinoma arising from a malignant struma ovarii. B) Depicts a serous cystadenoma with chronic inflammation and early fibrosis. CT, computed tomography

**Figure 2:**
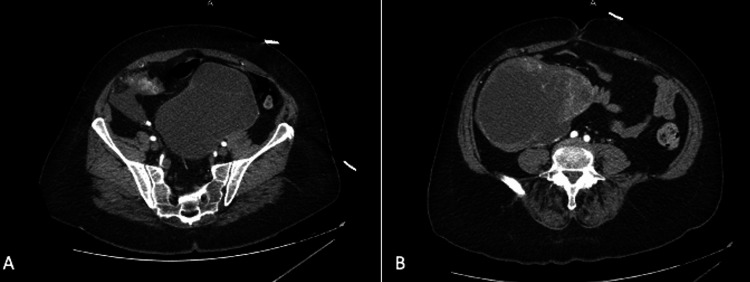
Axial images of CT scan showing bilateral ovarian masses. A) Depicts a serous cystadenoma with chronic inflammation and early fibrosis. B). Depicts the poorly differentiated thyroid carcinoma arising from a malignant struma ovarii. CT, computed tomography

A 58-year-old female presented to the Emergency Department with a history of chest congestion and progressive shortness of breath for one week. She also reported protracted nausea and vomiting for one day, with coffee-ground emesis occurring the day she presented to the hospital. Upon obtaining the history from the patient, she denied abdominal pain but did report difficulties with constipation that she had been self-managing for several years. Physical exam revealed 88% oxygen saturation, tachycardia, bilateral expiratory wheezes, a protuberant abdomen, hypoactive bowel sounds, right lower quadrant tenderness, right lower quadrant dullness to percussion, and a palpable abdominal mass at the right lateral border of the abdomen. A computed tomography (CT) of the chest, abdomen, and pelvis was ordered on the indication of hypoxia and gastrointestinal bleeding. While the CT revealed patchy infiltrates consistent with a bibasilar viral pneumonia, it incidentally revealed two large complex part cystic and part enhancing solid masses of the lower abdomen-pelvis region (Figures 1, 2). The right mass measured 10x13x12.6cm (Figure 1A) and the left-central mass measured 13x12.5x12.3cm (Figure 1B) according to the imaging. Based on imaging and size of the masses, origin was indeterminate but radiology deemed genitourinary neoplasm most likely. 

**Figure 3:**
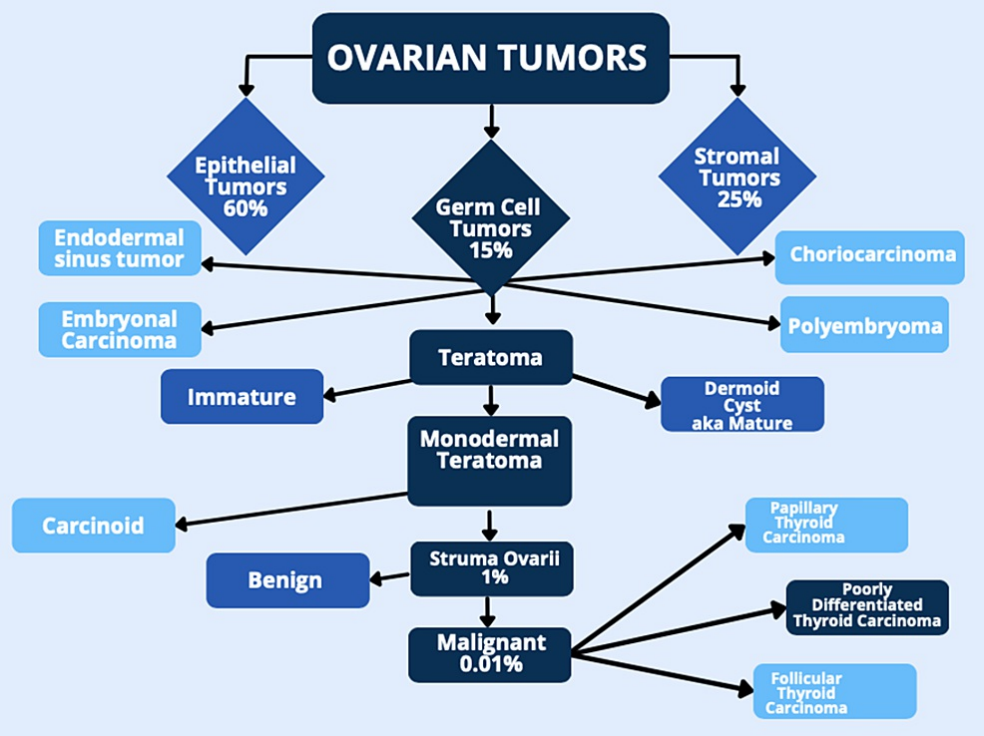
Classification and prevalance of ovarian tumors. Percentages depict the estimation of cases in comparison to all ovarian tumors. Example: Malignant struma ovarii make up 0.01% of all ovarian tumors. Image Credit: Jessie C. Harrison

The patient was admitted and both general surgery and gynecology were consulted. Gynecology consultation hypothesized the masses were secondary to epithelial ovarian carcinoma. The hypothesized diagnosis was sound due to the increased prevalance of epithelial tumors in comparison to germ cell or stromal tumors (Figure 3). Cancer antigen 125 protein (CA 125) was ordered, as it can monitor certain cancers before, during, and after treatment including ovarian cancer. CA 125 was elevated at 101 U/mL (reference value < 38 U/mL). Gynecology consultation originally advised that patient care be transferred to a gynecologic oncologist for resection of the masses and postoperative follow-up. However, this would have resulted in a transfer to another facility hours away. The patient chose to continue care with general surgery and was referred to a local oncologist for follow-up. General surgery suspected an ovarian tumor and agreed with the recommendation of mass removal due to the size and obstructive-like patterns in her abdomen. The patient underwent an exploratory laparotomy. Intra-operative findings include two excised ovarian masses measuring 15cm on the right and 19cm on the left, a portion of resected small bowel which was intimately connected to the right ovarian mass, and four quadrant lavage was obtained for cytology.

Pathology from the left oophorectomy confirmed a serous cystadenoma with patchy chronic inflammation and early fibrosis. The right showed PDTC arising in the background of struma ovarii with a cystic teratoma consisting of an epidermal cyst and adipose, vascular, and neural tissue. The initial impression of the right salpingo-oophorectomy appeared to be a primary ovarian neoplasm, however numerous structures contained eosinophilic material consistent with colloids. Sections from the right ovarian mass were sent to Mayo Clinic Laboratories for review. The pathologists concluded the tumor showed: 1) extensive solid-insular growth patterns with foci exhibiting invasion into the dense fibrous, pseudocapsular tissue surrounding the tumor and lymphatic spaces; 2) vascular invasion; and 3) focal comedo-type necrosis - all of which were consistent with PDTC. The Mayo Clinic also performed immunohistochemical panel for an MSO. Thyroglobulin, TTF-1, and PAX-8 were positive and Ki-67 was increased. Synaptophysin, chromogranin, SF-1, EMA, and CD-99 were negative.

**Figure 4:**
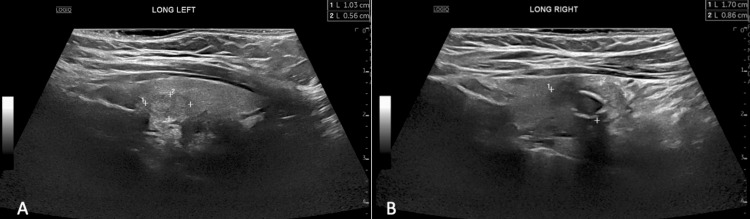
Thyroid gland ultrasound of left (a) and right (b) nodules, which pathology showed to be papillary thyroid carcinoma. A) Largest nodule in the left lobe measuring 1.2x1.0x0.9cm is hypoechoic with no associated calcifications. B) Largest right nodule measuring 1.7x0.8x0.9cm is hypoechoic with coarse peripheral calcifications.

After following up with Endocrinology, and two months after her exploratory laparotomy, the patient underwent a thyroid ultrasound which showed multifocal thyroid nodules (Figure 4). The largest nodule on the right lower pole measured 1.7x0.8x0.9cm with peripheral calcifications and the largest nodule on the left lobe measured 1.2x1.0x0.9cm with solid and cystic features. Endocrinology and general surgery agreed a total thyroidectomy was an appropriate management of the patient’s presentation. The total thyroidectomy was performed and sections were sent to pathology for review. Pathology confirmed two well-differentiated PTC of the thyroid gland that were 1.3 and 0.6cm in size. The carcinoma was confined to the thyroid, but did extend to the capsular margin in the left lobe lesion. No lymphovascular invasion was identified. Radiology determined the thyroid tumor was most likely a separate primary lesion, rather than the origin of metastasis. The patient received radioiodine ablation one month following her thyroidectomy. She was prescribed levothyroxine and monitored by her endocrinologist and oncologist. She reports neuropathy and mild memory impairment which she believes is attributed to the surgical management of her PDTC arising from struma ovarii and concurrent PTC, but otherwise has no complaints. She is currently scheduled for and awaiting a follow-up Positive Emission Tomography (PET) scan nine months after surgery and ablation.

## Discussion

Struma ovarii exhibits the same histological and physiological properties as that of the cervical thyroid tissue [5]. The average age of diagnosis is between 40 and 60 years old [4]. Malignant struma ovarii can be difficult to diagnose due to patient presentation and histomorphological features. Though patients can present clinically with abdominal mass, abdominal pain, menstrual irregularities, and hyperthyroidism, some can be asymptomatic [6]. Some ovarian masses have been detected due to incidental discovery during ordered imaging for other indications, as it was for our patient, or while performing gynecological surgery such as a unilateral salpingo-oophorectomy [7].

Malignant transformation of ovarian struma is extremely rare. It is estimated to have an incidence of less than 1 in 10 million women annually [8]. Malignancy is characterized by angioinvasion or gross invasion into adjacent tissue - our patient had both angioinvasion and invasion of the small bowel. In the setting of PDTC arising from MSO, this type of carcinoma is a prognostic intermediate between well-differentiated thyroid carcinomas and anaplastic carcinomas. Well-differentiated carcinomas like follicular thyroid carcinoma and papillary thyroid carcinoma are indolent, whereas anaplastic carcinoma is rapid growing and usually fatal [9]. PDTC diagnostic criteria, coined the Turin criteria, issued by an international group of pathologists is as follows: (1) solid, trabecular or insular pattern of growth, (2) conventional nuclear features of papillary carcinoma is absent, and (3) has at least one of the following features - convoluted nuclei, tumor necrosis, or high mitotic activity [10]. Our patient meets these criteria due to the solid/insular pattern of growth, absence of papillary nuclear features, and presence of tumor necrosis. Another diagnostic criteria for PDTC was issued by the Memorial Sloan Kettering Cancer Center (MSKCC) and used presence of tumor necrosis or high mitotic rate to diagnose - which still would be met by the patient in our case study due to the tumor necrosis [10]. There was no minimum percentage of PDTC components outlined in the criteria because it has been shown that even a small amount, such as 10%, will have an effect on prognosis. This is why a thorough histological analysis is necessary to ensure the correct diagnosis is made. PDTC has many possible routes to existence; it can arise de novo from a follicular epithelial cell or it can progress from either follicular or papillary carcinoma [9]. 

PDTC can be differentiated from other forms of ovarian carcinomas by immunohistochemical analysis. The presence of thyroglobulin can differentiate a struma ovarii from ovarian epithelial carcinoma or sex-cord stromal tumors histologically [2]. PDTC is usually positive for PAX-8, thyroglobulin, and TTF-1 [8], which was seen in this case report. Mainstay treatment for benign struma ovarii typically ends with resection of the tumors. However, treatment for non-metastatic malignant struma ovarii does not have a standard [11]. With so few cases reported or reviewed, often an aggressive approach of total thyroidectomy is performed, as seen in treatment of metastatic MSO [12,13]. Most physicians will also lean toward a radioactive iodine ablation following the thyroidectomy to use thyroglobulin levels as a tumor marker, which was also seen as the treatment provided for our patient [14].

## Conclusions

This is a rare case of a 58-year-old female who presented with a history of chronic constipation over several years and a palpable abdominal mass in the right lower quadrant. With the aid of CT imaging, exploratory laparotomy, ultrasound, thyroidectomy, immunohistochemical panels, and histopathological studies, the diagnosis of MSO and concurrent PDTC was confirmed. Our case report discusses the etiology, prevalance, clinical presentation, and management strategies of MSO with concurrent PDTC. PDTC arising in the background of malignant struma ovarii is extremely rare and often creates a diagnostic challenge for pathologists due to the histomorphological presentation of differential diagnoses. Immunohistochemical studies including thyroglobulin, TTF-1, and PAX-8 along with Turin’s criteria are essential in proper diagnosis, which leads to proper treatment. Since there are so few reported cases of MSO, let alone MSO with concurrent PDTC, there is no formal management. However, due to the risk of metastasis or recurrence, appropriate patient surveillance and post-operative follow-up is essential.
